# Distribution of Charged Residues Affects the Average Size and Shape of Intrinsically Disordered Proteins

**DOI:** 10.3390/biom12040561

**Published:** 2022-04-09

**Authors:** Greta Bianchi, Marco Mangiagalli, Alberto Barbiroli, Sonia Longhi, Rita Grandori, Carlo Santambrogio, Stefania Brocca

**Affiliations:** 1 Department of Biotechnology and Biosciences, University of Milano-Bicocca, 20126 Milan, Italy; greta.bianchi@unimib.it (G.B.); marco.mangiagalli@unimib.it (M.M.); rita.grandori@unimib.it (R.G.); 2 Departement of Food, Environmental and Nutritional Sciences, University of Milan, 20133 Milan, Italy; alberto.barbiroli@unimi.it; 3 Laboratory Architecture et Fonction des Macromolécules Biologiques (AFMB), UMR 7257, Centre National de la Recherche Scientifique (CNRS), Aix Marseille University, 13288 Marseille, France; sonia.longhi@univ-amu.fr

**Keywords:** charge clustering, polyelectrolytes, average shape of conformational ensembles, charged-residue patterning, hydrodynamic radius, solvent-accessible surface area, proline content, conformational compactness, ellipsoid model

## Abstract

Intrinsically disordered proteins (IDPs) are ensembles of interconverting conformers whose conformational properties are governed by several physico-chemical factors, including their amino acid composition and the arrangement of oppositely charged residues within the primary structure. In this work, we investigate the effects of charge patterning on the average compactness and shape of three model IDPs with different proline content. We model IDP ensemble conformations as ellipsoids, whose size and shape are calculated by combining data from size-exclusion chromatography and native mass spectrometry. For each model IDP, we analyzed the wild-type protein and two synthetic variants with permuted positions of charged residues, where positive and negative amino acids are either evenly distributed or segregated. We found that charge clustering induces remodeling of the conformational ensemble, promoting compaction and/or increasing spherical shape. Our data illustrate that the average shape and volume of the ensembles depend on the charge distribution. The potential effect of other factors, such as chain length, number of proline residues, and secondary structure content, is also discussed. This methodological approach is a straightforward way to model IDP average conformation and decipher the salient sequence attributes influencing IDP structural properties.

## 1. Introduction

Intrinsically disordered proteins (IDPs) and regions have a biased sequence composition compared to folded counterparts, being enriched in disorder-promoting and charged amino acids and depleted in order promoting ones [1,2,3]. The high number of charged residues (Asp, Glu, Arg, Lys) has enabled modeling IDPs as either polyelectrolytes or polyampholytes, depending on the presence of same- or opposite-sign charges, respectively. The charge state of polyampholytes is often described by the total fraction of charged residues (*FCR*), obtained as the sum of the fractions of positive (*f_+_*) and negative residues (*f_−_*), and by the net charge per residue (*NCPR*), calculated as the difference between *f_+_* and *f_−_* [4]. In addition to these coarse-grain parameters, the linear distribution of positive and negative charges, described by *κ* or sequence charge decoration parameters [5,6], is also an important feature in determining protein compactness. More in detail, computational and experimental data show that charge segregation promotes protein compaction [7,8,9,10].

IDPs consist of fluctuating and interconverting conformations that constitute “conformational ensembles”. Size-exclusion chromatography (SEC), which enables molecule separation based on their hydrodynamic radius (*R_h_*), is one of the most popular and easy to apply techniques to study the compaction of proteins, including IDPs. Experimentally, *R_h_* can be determined from the chromatographic elution volume, using a calibration curve obtained with proteins of known *R_h_*, or known molecular mass belonging to the same structural class [11,12]. Achieving a more quantitative description of IDP ensembles requires methods capable of dealing with heterogeneous molecular systems, such as nuclear magnetic resonance (NMR), small-angle X-ray scattering (SAXS), mass spectrometry (MS) combined with labeling techniques, high-speed atomic force microscopy, and Förster resonance energy transfer and non-denaturing mass spectrometry (native MS) to cite a few [13,14,15,16,17]. Native MS has been extensively employed to characterize the properties of heterogeneous conformational ensembles, enabling the detection of even poorly populated states [18,19,20,21]. Indeed, gentle ionization conditions, such as those obtained by nano-electrospray ionization (nanoESI), preserve non-covalent interactions under the vanishing-solvent conditions of the electrospray, leading to protein ionization and transfer to the gas phase. The final protein net charge is mainly dictated by structural compactness under controlled conditions. Thus, charge state distributions (CSDs) in nanoESI spectra reveal the main components of conformational ensembles [17,20,22]. Unfolded/disordered proteins achieve higher charge states than their globular counterparts. For both folded and unfolded chains, the average charge state correlates with the solvent-accessible surface area (*SASA*), reflecting chain compactness [17,23,24,25].

**Figure 1:**
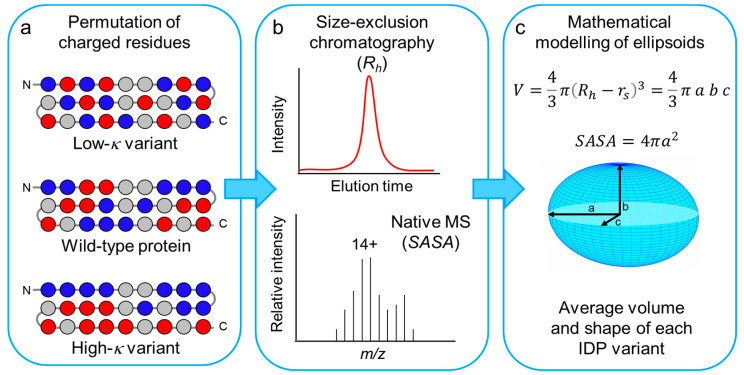
Scheme of the experimental plan used in this work. (**a**) Scheme of the primary structures of a protein set, derived from a generic wild-type IDP by distributing more evenly the oppositely charged residues (low-*κ* variant) or by clustering them in two blocks at the N- and C-moieties (high-*κ* variant). Only charged residues were permutated, preserving the original location in the sequence of non-charged residues (see also Figure S1). Blue and red spheres indicate positively and negatively charged residues, respectively. Gray spheres indicate all the other amino acid residues. (**b**) The conformational ensemble of each model IDP was investigated by size-exclusion chromatography (SEC) and native mass spectrometry (MS), to derive experimental values of *R_h_* and *SASA*. (**c**) *R_h_* and *SASA* values were combined to calculate the volume and depict the average shape from the ensemble of each model IDP.

In spite of the seminal and breaking-through studies by Pappu and co-workers that illuminated the relationships between charge distribution and conformational properties of IDPs [7,8,10], a full understanding of how the sequence of IDPs encodes their conformation is still lacking, thereby preventing, for instance, the *ex nihilo* design of IDPs with a precise set of desired conformational properties. With the goal of shedding light on these still unsolved issues, here we have studied the effect of charge segregation on three model IDPs that exhibit similar content in overall charged residues, net charge, and hydropathy, but different content of proline residues and secondary structure, and slightly different size. Charged residues within these model IDPs were permutated to obtain different *κ*-variants (Figure 1), and the three sets of proteins were characterized by SEC and ESI-MS. Experimentally derived *R_h_* and *SASA* values were used to obtain coarse-grained structural information on these IDP ensembles using a recently published model, originally developed for globular proteins, that approximates the geometry of a protein to an ellipsoid [26].

Results show how the changes in average volume and shape triggered by the distribution of charged residues are variously affected by the frequency of proline residues. In addition, we discussed the potential role of other factors such as secondary structure content and amino acid chain length.

## 2. Materials and Methods

### 2.1. Gene Design and Cloning

The model molecules employed in this study are IDPs derived from the measles virus N protein, N_TAIL_ [27], from the Hendra virus P protein, PNT4 [28], and from the human medium neurofilament protein, NFM (UniProtKB ID: P07197) [29,30]. The region used in this work (residues 790–916) belongs to the KE-rich tail of NFM, which is predicted to be intrinsically disordered. The rules followed for NFM gene design are those used for PNT4 and N_TAIL_ [8]. Briefly, we conceived low-*κ* and high-*κ* variants sharing with wild type (wt) the same number of charged residues and the same position of non-charged residues and differing just in the distribution of positively (Lys, Arg) and negatively (Glu, Asp) charged residues along the sequence. In high-*κ* sequences, positively and negatively charged residues are clustered in the N- and C-terminal regions, respectively. On the contrary, in low-*κ* sequences, positively and negatively charged residues are more evenly distributed than in the wt sequence. Synthetic genes encoding for NFM were optimized for expression in *Escherichia coli* (Genscript, Piscataway, NJ, USA) and cloned into the pET-21a vector (EMD, Millipore, Billerica, MA, USA) between the *Nde*I and *Xho*I sites (Jena Biosciences, Jena, Germany). Each synthetic gene encodes a protein with an N-terminal hexa-histidine (6xHis) tag, while a stop codon has been inserted immediately before the *Xho*I restriction site, thereby excluding from the coding region the vector-encoded 6xHis tag. The amino acid sequences are shown in Figure S1. *Escherichia coli* DH5α™ strain (Invitrogen, Waltham, MA, USA) was used for plasmid DNA propagation.

### 2.2. Production and Purification of κ Variants

The *E. coli* strain BL21 (DE3) (EMD Millipore, Billerica, MA, USA) was used for protein heterologous production. Cultures were grown in ZYM-5052 medium [31], and recombinant IDPs were extracted and purified as described by Tedeschi and co-authors [9]. Briefly, recombinant proteins were purified from the soluble fraction of the bacterial lysate by gravity-flow, immobilized-metal affinity chromatography using a nickel-nitrilotriacetic acid agarose resin (ABT, Torrejon de Ardoz, Madrid, Spain). The fractions exhibiting the highest concentration were pooled, and buffers were exchanged for phosphate-buffered saline (PBS, 150 mM NaCl, 50 mM sodium phosphate, pH 7.0) or ultrapure ammonium acetate buffer (ammonium acetate 50 mM, pH 6.95, Merck KGaA, Darmstadt, Germany) by gel filtration on PD-10 columns (GE Healthcare, Little Chalfont, UK). Protein concentration was determined by Bradford protein assay (Bio-Rad, Hercules, CA, USA), using bovine serum albumin as a standard.

### 2.3. Bioinformatics Analysis

Sequence analysis of model proteins was performed using CIDER [32] and IUPred [33] web servers. IUPred provides a score that characterizes the disordered tendency of each position along the sequence. The score ranges from 0 to 1, with predicted scores above 0.5 indicating disorder. CIDER was used with default parameters to compute *κ* values and local sequence properties such as *NCPR*, *FCR*, and the mean hydrophobicity in the 0–9 scaled Kyte-Doolittle hydropathy score.

### 2.4. Far-UV Circular Dichroism (CD) Spectroscopy

Far-UV CD analyses were carried out in PBS using a Jasco J-815 spectropolarimeter (Jasco Europe, Lecco, Italy) in a 1-mm path-length quartz cuvette. Measurements were performed at variable wavelengths (190–260 nm) with a scanning velocity of 20 nm/min and a data pitch of 0.2 nm. All spectra were corrected for buffer contribution, averaged from three independent acquisitions, and smoothed by the Means-Movement algorithm implemented in the Spectra Manager package (Jasco Europe, Lecco, Italy). Experiments were performed in triplicate. Mean ellipticity values per residue ([θ]) were calculated as described by Tedeschi and co-authors [9]. The deconvolution of CD spectra to assess secondary structure content was performed using the BestSel program [34].

### 2.5. Analytical SEC

Recombinant IDPs produced in this work were analyzed by SEC within the day they were purified. Chromatographic separations were carried out on a Superose 12 10/300 GL column (GE Healthcare, Milan, Italy), in mobile phase PBS, at a flow rate 0.5 mL/min. The chromatographic system was composed of a Waters Delta 600 pump, a 600 Controller, and a 2487 Dual λ Absorbance Detector; all managed through the Empower Pro Software (Waters Corporation, Milford, MA, USA). Chromatograms were recorded at 220 nm. The calibration curve was built using the following standards: Apo-ferritin (horse spleen, 443 kDa, *R_h_* 6.1 nm), Alcohol dehydrogenase (yeast, 150 kDa, *R_h_* 4.6 nm), BSA (bovine serum, 66 kDa, *R_h_* 3.5 nm), Ovalbumin (chicken egg, 43 kDa, *R_h_* 2.8 nm), Carbonic anhydrase (bovine erythrocytes, 29 kDa, *R_h_* 2.1 nm), Cytochrome C (horse heart, 12.4 kDa, *R_h_* 1.7 nm [35].

Firstly, for each standard protein the distribution coefficient (*K_d_*) was calculated:

(1) $${K_{d}}_{\;}=\frac{V_{e}-V_{0}}{V_{t}-V_{0}}$$

where *V_e_* is the elution volume, *V*_0_ the void volume, and *V_t_* the total volume. Uracil (0.112 kDa) and Blue dextran (2000 kDa) were used for *V_t_* and *V*_0_ determination.

Finally, the calibration curve Log(*R_h_*) vs. *K_d_* was built and the interpolated linear equation used to calculate IDPs hydrodynamic radii from their *K_d_* values. IDPs were run at least in triplicate.

The theoretical radius (*R_t_*) was calculated according to the empirical Equation (2) [36].

(2) $$R_{t}=\left(1.24\;P_{pro}+0.904\right)\;\left(0.00759\;\left|Q\right|+0.963\right)\;S_{his*}$$

where *P_pro_* is the number of proline residues, |*Q*| the absolute net charge and the *S_his__∗_* is 0.901 or 1 depending on whether a 6xHistag is present or absent, respectively.

*R_h_* values were used to calculate the compaction index (*CI*), which provides a simple and continuous descriptor useful for comparing conformational properties of IDPs of different lengths [23,37]. The *CI* derived from the experimental value of *R_h_* (*CI_R_*) was calculated by applying the following equation [37]:

(3) $$CI_{R}=\frac{R^{D}-R_{h}}{R^{D}-R^{NF}}$$

where *R_h_* is the experimental value, *R^D^* and *R^NF^* are the theoretical values of a chemically denatured or a folded protein, calculated on the basis of power-law Equations (4) and (5), which describe their dependence on the number of residues, *N* [11].

(4) $$R^{NF}=4.92\cdot N^{0.285}$$

(5) $$R^{D}=2.49\cdot N^{0.509}$$

### 2.6. Native MS Analyses

Protein solutions in 50 mM ammonium acetate, pH 7.0, were brought to a concentration of 10 µM, and samples under non-denaturing conditions were directly injected at room temperature into an Orbitrap Fusion mass spectrometer (Thermo Fisher Scientific, Waltham, MA, USA) equipped with a nano-electrospray ion source. Metal-coated borosilicate capillaries with medium-length emitter tips of 1 μm internal diameter were used to infuse the sample. To assess the effect of electrostatic interactions, protein samples were also analyzed at higher ionic strength (200 mM ammonium acetate pH 7.0) and low pH (no buffer, 1% formic acid, pH 2.5). The following instrumental setting was applied: ion spray voltage, 1.1–1.2 kV; ion-transfer tube temperature, 275 °K; AGC target, 4 × 10^5^; maximum injection time, 100 ms. Spectra were averaged over 1-min acquisition. Multi-Gaussian fitting of MS spectra was performed employing the program OriginPro 2020 (OriginLab Corporation, Northampton, MO, USA), and *CI* of single conformers ($CI_{SASA}^{i}$) and ensembles (${\bar{CI}}_{SASA}$) were calculated as follows [16]:

(6) $$CI_{SASA}^{i}\;=\frac{A^{c}-A^{0}}{A^{c}-A^{f}}$$

(7) $${\bar{CI}}_{SASA}\;=\sum_{i=1}^{n}w_{i}.^{\;}CI_{SASA}^{i}$$

where *A^c^* and *A^f^* are the solvent-accessible surface areas derived by native MS for reference, random coil (*c*) and folded (*f*) proteins of the same size of the protein under study, *A*^0^ is the solvent-accessible surface areas derived by native MS for the conformer (exploiting the charge state—*SASA* relationship), *w_i_* is the relative amount of the conformer with compaction index $CI_{SASA}^{i}$.

Statistical significance of experimental differences was estimated by performing a Welch’s *t*-test on three independent datasets.

### 2.7. Application of Ellipsoid Model

The ellipsoid model assumes that the average conformation of a given protein can be represented by an ellipsoid with semi-axes *a*, *b,* and *c* (*a* ≥ *b* ≥ *c*) [26]. The experimental ellipsoid volume depicting the conformation of the IDP averaged over the ensemble can be estimated by the volume of a sphere given by the following formula:

(8) $$V=\frac{4}{3}\pi {\left(R_{h}-r_{s}\right)}^{3}$$

where *r_s_* represents the hydration shell (generally assumed to be 5 Å) [38,39], and *R_h_* the hydrodynamic radius obtained by SEC experiments. The geometrical volume of an ellipsoid is expressed as:

(9) $$V=\frac{4}{3}\pi abc,$$

To calculate *a*, the quadratic relationship with *SASA* given by the model of Wu and co-authors [26] can be exploited:

(10) $$SASA=4\pi a^{2},$$

Then, *b* and *c* values can be approximated by weighted averages between the extreme conditions of prolate (*a* > *b* = *c*) and oblate (*a* = *b* > *c*) ellipsoids, according to the equations published by Wu and co-authors [26].

Thomsen’s approximation was employed to calculate the ellipsoid surface area (maximal discrepancy to real surface ~1%).

The ellipsoid *flattening* was described through the values of *f_b_* and *f_c_*, calculated according to the formulas:

(11) $$f=\frac{\left(a-b\right)}{a};\;f=\frac{\left(a-c\right)}{a}$$

Both indices report the eccentricity of axial elliptic sections of the ellipsoid, and span in the range [0;1), where 0 corresponds to a circular section.

## 3. Results

### 3.1. Design of Model IDPs by Permutation of Charged Residues

The model IDPs used in this work are the viral proteins PNT4 and N_TAIL_ and a C-terminal IDR from the human NFM. These IDPs are similar in length, theoretical hydrodynamic radius (*R_t_*), charge density, and charge segregation, as witnessed by their *κ* value (Table 1). Values of *κ* vary between 0 and 1, with 0 indicating evenly mixed positive and negative residues, and 1 referring to the complete segregation of oppositely charged residues along the linear sequence [4]. In our model proteins, the number of positive and negative charges is well balanced, producing a rather low *NCPR* (mean absolute value 0.038 ± 0.017), and opposite charges are evenly distributed along the sequence, thereby resulting in rather low *κ* values (mean value 0.167 ± 0.041). The three proteins differ in the fraction of proline residues, which is 0.7%, 5.2%, and 11.4% for NFM, N_TAIL_, and PNT4, respectively. Among disorder-promoting residues, proline residues are also recognized to disfavor α-helical and β-structures [40], and to promote extended conformations by conferring rigidity to the backbone [36]. For each model IDP, a “high-*κ*” and a “low-*κ*” variants were designed by permuting charged residues while keeping the position of all other residues unchanged. Table 1 summarizes, for each model protein and its variants, the κ parameter, *NCPR*, and *FCR* values calculated using the CIDER webserver [28]. 

**Figure 2:**
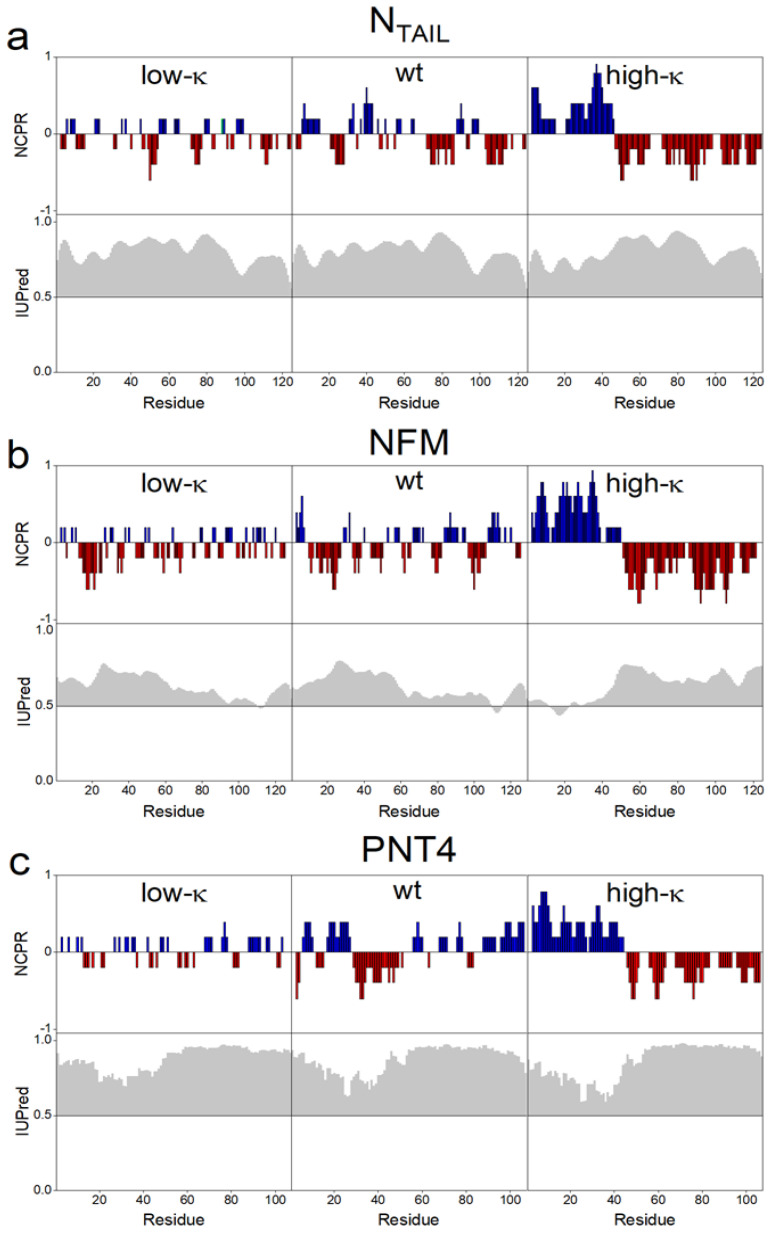
Comparative bioinformatic analyses of N_TAIL_ (**a**), NFM (**b**) and PNT4 (**c**). Upper panels: The *FCR*, fraction of charged residues, was calculated by CIDER [32]. Each model protein contains charged residues at high density, with red and blue bars indicating negative and positive charges, respectively. The increase in *κ* value is reflected in the progressively more “blocky” distribution of charged residues. Lower panel: each protein is predicted to be predominantly disordered by IUPred [33]. The discrepancy from the disorder threshold value (0.5) in the IUPred score is shaded in gray. The IUPred and CIDER outputs were generated using the default options of the respective web server.

In the high-*κ* variants, positive and negative charged residues are clustered in two distinct blocks at the N- and C-terminal moieties of the sequence, while in low-*κ* variants, these residues are evenly alternated along the sequence, as highlighted by their *NCPR* profiles (Figure 2a–c, upper panels, and Figure S1). The degree of disorder predicted by IUPred [33] is conserved within each set of model proteins derived by permutation from the respective wt sequence (Figure 2a–c, lower panels). The three sets of proteins were recombinantly produced and purified by immobilized-metal affinity chromatography and experimentally assessed by CD analysis in the far-UV (Figure S2). The CD spectra of wt IDPs display the typical trait of structural disorder with a negative peak at ~200 nm (black line in Figure S2). Worthy to note, all the spectra of wt IDPs present a small shoulder at ~220 nm, which indicates the presence of some elements of helical secondary structure. Despite the common high level of disorder predicted by IUPred, deconvolution of CD spectra indicates that in all the three model IDPs the α-helical content tends to increase along with the values of *κ* (Figure S2, inset).

### 3.2. Impact of Charge Clustering on the R_h_ of the Model IDPs

**Figure 3:**
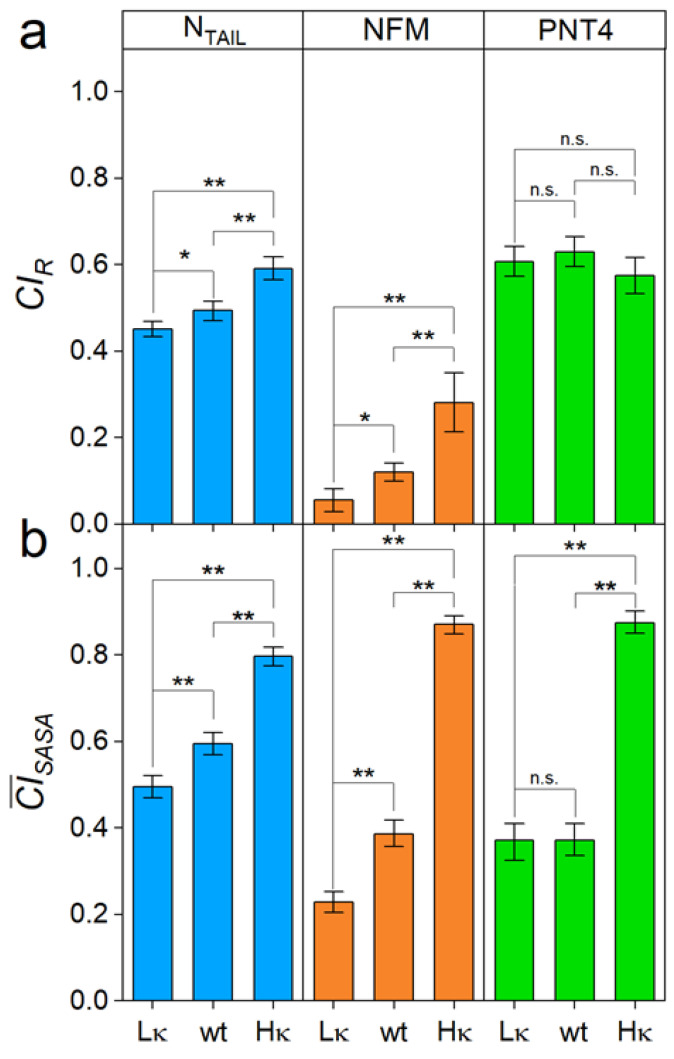
Compactness of the model IDPs. (**a**) *CI* derived from the *R_h_* (*CI_R_*); (**b**) *CI* derived from the average *SASA* of the conformational ensemble (${\bar{CI}}_{SASA}$) of N_TAIL_, NFM and PNT4 variants (L*κ*: low-*κ*; wt: wild type; H*κ*: high-*κ*). Mean values of three independent measurements are shown with error bars indicating standard deviations. Statistical analyses were carried out using Welch’s *t*-test, n.s.: not significant *p* > 0.05, *: *p* < 0.05, **: *p* < 0.01.

Size-exclusion chromatography was employed to estimate the *R_h_* values of the three sets of model IDPs (Table 2). Experimental *R_h_* values of wt N_TAIL_ and wt PNT4 (2.71 ± 0.09 and 2.34 ± 0.11 nm, respectively) are close to the theoretical ones (Table 1) and similar to the previously determined ones [9]. The *R_h_* of wt NFM (3.31 ± 0.12) is determined here for the first time. We observed that *R_h_* decreases as *κ* increases for N_TAIL_ and NFM, but not for PNT4 (Table 2). To compare the compaction properties of IDPs with different chain lengths, *R_h_* data were used for the calculation of the *R_h_*-based *CI* (*CI*_R_, defined in Equation (2)). The value of *CI* ranges from 0 to 1, corresponding to minimal and maximal compaction, respectively [37]. Analysis of the *CI*_R_ confirms that N_TAIL_ and NFM significantly respond to charge segregation, while PNT4 average compactness is not affected by the *κ* value (Figure 3a).

### 3.3. Impact of Charge Clustering on the Conformational Ensemble of the Model IDPs

**Figure 4:**
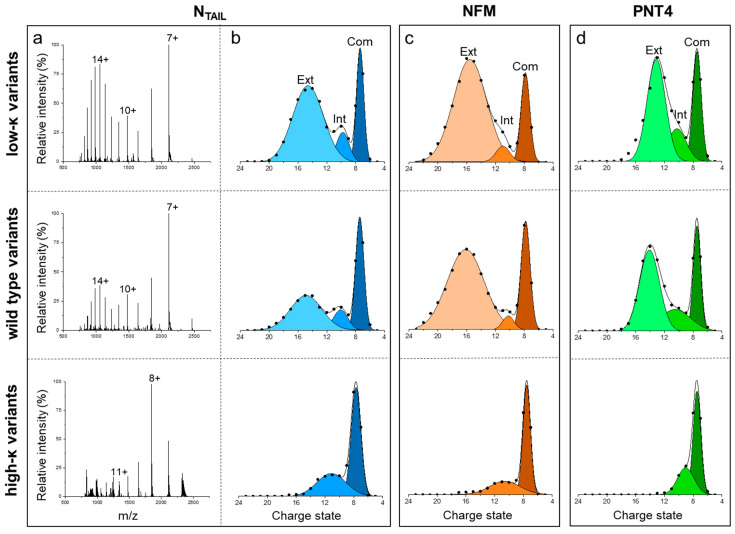
Native-MS analyses. (**a**) NanoESI-MS spectra of N_TAIL_ variants acquired under non-denaturing conditions (50 mM ammonium acetate pH 7.0). The most intense signal of each peak-envelope is labeled by the corresponding charge state. (**b**–**d**) Multi-Gaussian deconvolution of the MS spectra obtained for N_TAIL_ (**b**), NFM (**c**) and PNT4 (**d**), in the low-κ (upper row), wt (central row) and high-κ (bottom row) variants. Extended (Ext), intermediate (Int) and compact (Com) species are colored with different shades and labeled in the upper panels. MS spectra of NFM and PNT4 variants are reported in Figure S3.

Native MS was employed to assess the conformational properties of the three sets of IDPs. In this approach, the CSDs resulting from the nanoESI process reflect the overall compactness and relative amounts of the main conformers in the original solution [17,18,22]. Native-MS spectra obtained under non-denaturing conditions for the three variants of N_TAIL_ (Figure 4a), NFM, and PNT4 (Figure S3) display multimodal CSDs, highlighting the heterogeneous conformational ensemble typical of IDPs. Multi-Gaussian deconvolution of the MS spectra of the wt IDPs (Figure 4b–d, central row) indicates that these variants exist in three main conformational components. For each component, the *SASA* and the corresponding *CI* ($CI_{SASA}^{i}\;$defined in Equation (7)) were calculated as recently described [17]. The components were classified as “extended” ($CI_{SASA\;}^{i}$< 0.25), “intermediate” (0.25 < $CI_{SASA}^{i}$ < 0.75), and “compact” ($\;CI_{SASA}^{i}$> 0.75) (Figure S4). In all the model IDPs, the three main conformational components observed in the wt IDPs also characterize the ensemble of low-*κ* variants, but not that of high-*κ* variants, which includes only the “intermediate” and “compact” components (Figure 4). These data indicate that charge clustering induces a loss of heterogeneity of conformational components, in favor of more compact states, in agreement with the increase in secondary structure observed by CD spectroscopy on our model proteins and also with results obtained on p27 by ion-mobility MS [8]. To gain a more comprehensive view of charge clustering effects on IDP conformation, we calculated the *CI* based on the average *SASA* (${\bar{CI}}_{SASA}$), which weights the $CI_{SASA}^{i}\;$(Figure S4) by the relative abundance (Figure S5) of the corresponding conformational component. The analysis of ${\bar{CI}}_{SASA}$ indicates that the protein compactness increases with *κ* (Figure 3b). These results are in good agreement with those obtained by SEC, confirming the general trend of protein compaction at increasing *κ* values and the peculiar behavior of PNT4. In this latter case, the ${\bar{CI}}_{SASA}$ does not vary for low-*κ* and wt variants, and it strongly increases just for high-*κ* variants (Figure 3). Overall, the largest differences between MS and SEC results are obtained for the high-*κ* variants. To rule out possible technical artifacts, control MS experiments were carried out, exposing high-*κ* variants to acidic pH (formic acid 1%, pH 2.5) or higher ionic strength (ammonium acetate 200 mM). Indeed, electrostatic interactions are expected to be attenuated by the extensive protonation of all ionizable groups under very low pH conditions or by the charge shielding by salt ions. The resulting spectra show an increased amount of the components at high charge states, indicating that protein compaction is actually driven by in-solution electrostatic interactions (Figure S6).

### 3.4. Average Shape of the Model IDPs

The geometric, ensemble-averaged shape of each protein under investigation was predicted by combining the results for *R_h_* and *SASA*, as reported by Wu and co-authors [26]. The model was originally applied to approximate the shape of globular proteins to an ellipsoid, whose elongation (prolate-shaped) and/or flattening (oblate-shaped) describe the protein conformational transitions. The volume of the ellipsoid can be estimated from the experimentally derived *R_h,_* through Equation (8). By collating Equations (8) and (9), one obtains:

(12) $$V=\frac{4}{3}\pi abc=\frac{4}{3}\pi {\left(R_{h}-r_{s}\right)}^{3}$$

The average length of the *a*-axis was calculated through Equation (10), while the length of the *b* and *c* axes were obtained as described by Wu and co-authors [26].

**Figure 5:**
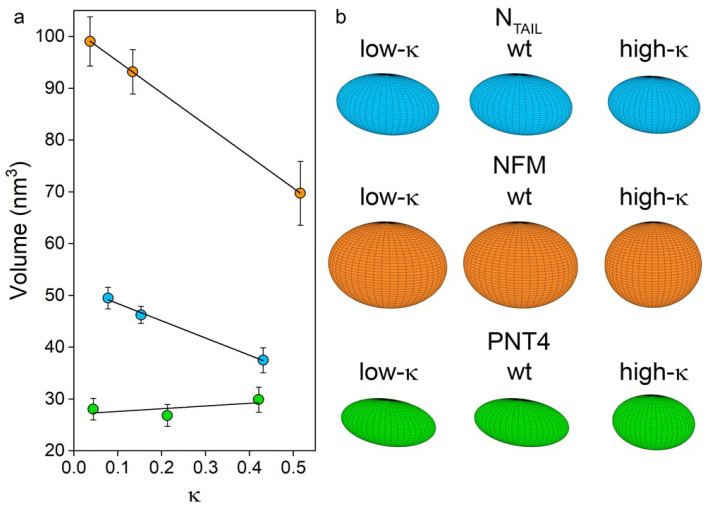
Relationship between ellipsoid volume and *κ* values. (**a**) Regression of ellipsoid volume and *κ* for N_TAIL_ (light blue), NFM (orange) and PNT4 (green). The equation of trend lines are: y = −33.4 x + 51.7, R^2^ = 0.987 for N_TAIL_, y = −61.2 x + 101.4, R^2^ = 0.998 for NFM and y = 4.3 x + 27.2, R^2^ = 0.710 for PNT4. Mean values of three independent measurements are represented, with error bars indicating standard deviations. (**b**) Geometry of the model proteins as obtained by applying the ellipsoid model.

The application of this model to the nine IDPs under investigation resulted in the values shown in Table 2 and Table S1 and represented in Figure 5, in which ellipsoid volumes and shapes are related to *κ* values. Comparing wt variants, NFM has the largest volume, followed by N_TAIL_ and PNT4. Considering the effects induced by charge clustering, and therefore moving from the lowest towards the highest *κ* values, a clear linear and negative correlation can be observed in the case of NFM and N_TAIL_ (overall reduction in volume of ~30% and ~25%, respectively) (Figure 5, Table 2). On the other hand, a neglectable effect was observed in the case of PNT4, for which the volume remains almost constant among the three variants, reflecting little variation of their *R_h_* value.

The shape of an ellipsoid depends on the length ratio of the *a*, *b,* and *c* axes, which in turn was derived from the experimental data of *SASA* (Table 2, Figure 3). The shape of an ellipsoid can be described by flattening indices (i.e., *f_b_* and *f_c_*), which report the eccentricity of axial elliptic sections. These indices vary in the range [0; 1), where 0 corresponds to circle sections, while elliptic sections of increasing eccentricity are obtained as the index approaches 1 (Table 2). Comparing wt variants, NFM has the most spherical conformation, followed by N_TAIL_ and PNT4 (which has the most prolate ensemble). As the *κ* value increases, the spheroid reshaping reflects the trends observed by native MS and reported in terms of ${\bar{CI}}_{SASA}$ with N_TAIL_ experiencing the smallest changes, and NFM and PNT4 the greatest ones (Table 2, Figure 3b). Indeed, on the basis of the flattening indices, the oblateness of N_TAIL_ is not significantly affected by *κ*, while NFM and PNT4 tend to approach a spherical shape.

## 4. Discussion

Computational and experimental works have already shown that charge clustering causes an overall increase in protein conformational compactness [7,8,9,10]. However, few data are available in terms of quantitative description of various conformational components within a heterogeneous ensemble. Our work highlights that the conformational ensembles of IDPs can be experimentally dissected by native MS to capture components of different *SASA* and abundance. Our results show that charge segregation triggers a loss of heterogeneity of conformational components, in favor of more compact and intermediate states. At the same time, we used SEC to monitor the average *R_h_* and observed an overall shrinkage resulting from charge clusterization.

To integrate the two kinds of information resulting from MS and SEC, and to obtain coarse-grained information on the shape of IDP ensembles, we applied a recently published model, which approximates the shape of globular proteins to ellipsoids [26]. The applicability of this “ellipsoid model” to IDPs, herein explored for the first time, is supported by three observations: (i) the relationship between CSD and *SASA* was proved to be independent of the folded or disordered nature of the proteins [20,23,25]; (ii) the ellipsoid model was successfully applied to depict the conformational changes induced by denaturation [26]. The broad molecular mass range of globular proteins for which the model was shown to hold true (i.e., ~9 kDa to ~70 kDa) [26] argues for the applicability of this model to the three model IDPs herein investigated whose mass falls within this range.

This model substantially helped us in translating and rationalizing the conformational effects induced by charge clustering into the shrinkage and loss of oblateness of each IDP ensemble, while providing evidence of singular, protein-specific compaction behaviors. The observation that each ellipsoid undergoes volume and shape changes in a protein-specific manner argues for a multifactorial response to charge segregation. Although referring to a small set of proteins, and hence likely not directly generalizable to all IDPs, our data suggest that proline content, chain length, and secondary structure content are potentially all involved in the response to charge segregation.

Proline content appears to play a relevant role in modulating the average conformational properties of the ensemble. Indeed, the abundance of proline residues (PNT4 > N_TAIL_ > NFM) promotes the ellipsoid oblateness in wt variants and counteracts the volume shrinking induced by *κ*. This is in line with the observations that proline disfavors α- and β structures [36,41] because of the conformational constraints imposed by its pyrrolidine ring [42] and the higher stiffness conferred by the preference towards the trans conformation of the Xaa-Pro peptide bonds [36]. Our data show that an increase from 0.7 to 5.2%, and then to 11% in proline content causes a significant reduction in the compaction response associated with charge clustering. Remarkably, the mean frequency of proline residues is 4.57 ± 0.05 and 8.11 ± 0.63 in databases of structured (i.e., PDB Select 25 [43]) and disordered proteins (i.e., DisProt [44,45]), respectively. In this scenario, proline residues would strongly hinder compaction driven by electrostatic interactions and reduce IDP propensity for induced folding. This indirectly supports the hypothesis that a high proline content is a compositional trait typical of “unfoldable IDPs”, in contrast to IDPs prone to undergo induced folding, which instead exhibit, at least locally, compositional features nearly overlapping with those of folded proteins [2,36,46]. This hypothesis is corroborated by the analyses of large protein datasets [46].

Polypeptide length may also affect the ellipsoid oblateness in wt variants and counteract *κ*-induced volume shrinking. Indeed, PNT4 (the shortest protein under investigation) responds to increasing *κ* with small volume changes and pronounced shape remodeling (from highly prolate ellipsoid to a more spherical geometry in the high-κ variant), whereas NFM (the longest chain herein studied) shows the greatest volume excursion among variants. Unfortunately, it is difficult to disentangle the contribution of chain length and proline content to charge clustering responsiveness: indeed, the attempt at rationalizing our experimental data and at dissecting the effect of protein length is hampered by the fact that PNT4 has the highest fraction of proline residue and NFM the lowest among our model proteins, thus making the effect of size and proline content overlapping.

Finally, the role of secondary structure content appears controversial. For each of the three proteins, charge clustering triggers an increase in the α-helical content. This could be related to the loss of heterogeneity among conformational components in favor of more compact and intermediate states observed by MS experiments. However, α-helical content does not correlate with compaction in terms of *CI_R_* and volume shrinkage (e.g., PNT4). This behavior seems to be consistent with previous studies indicating that the propensity of IDPs for compactness, unlike that of globular proteins, is not correlated with α-helical content [36,47]. Unfortunately, the paucity of data concerning the effects of charge segregation on IDP secondary structure makes it difficult to detail trends and deserves more extensive and systematic study.

Overall, our experimental data, complemented by the ellipsoid model, indicate that the extent of compaction and shape remodeling triggered by charge separation is modulated by multiple parameters that can concur, either individually or collectively, to counteract the expected response. Among the possible sequence features affecting IDP conformational responsiveness to charge clustering, the Lys/Arg and Asp/Glu ratio, recently reported by Zeng and co-authors [48], is a plausible factor that deserves further investigation. Many additional ones are probably at play and still remain elusive, thereby preventing our ability to fully rationalize and model the conformational behavior of IDPs.

## 5. Conclusions

In summary, the effect of charge segregation on the conformational properties of IDP ensembles was studied by applying a mathematical model that integrates experimental data from two orthogonal techniques, i.e., SEC and native MS. This original approach was proved to be more informative compared to the single techniques, delineating a distinct and protein-specific compaction behavior in terms of the size and shape of each conformational ensemble. The structural information afforded by this approach relies on techniques that are more accessible compared to more elaborate techniques, such as ion mobility, NMR, or SAXS, usually applied for the study of IDP ensembles. Potentially transposable on a larger scale, i.e., by using available experimental datasets of *SASA* and *R_h_*, this approach could also serve as an asset to a more systematic study of the individual factors influencing the compaction behavior of IDPs triggered by charge segregation.

Although we do not pretend to extend our findings to all IDPs, our work identified proline content, protein size, and intrinsic content in ordered secondary structure as factors governing IDP responsiveness. We hope that the present study will stimulate and foster future studies aimed at a systematic analysis of the elements that contribute to the conformational behavior of IDPs in response to charge clustering. In addition to unraveling the physicochemical rules underlying the response to charge segregation, these elements may account for sequence-specific and biologically relevant properties of proteins, such as the propensity to undergo induced folding or to exhibit partner-mediated conformational polymorphism. The next challenge will be to decipher the hierarchy of elements governing IDP conformation and how they can be modeled to better predict IDP behavior.

## Figures and Tables

**Table 1:** Features of the three model proteins and their derived *κ* variants. Sequence features were computed using CIDER [28]; the theoretical radius *R_t_* was calculated according to Marsh and Forman-Kay [35].

Protein	Number of Residues	Number of Prolines	Mean Hydropathy	*FCR*	*NCPR*	*R_t_* (nm)	*κ*	Variant
N_TAIL_	134	7	3.35	0.299	−0.045	2.64	0.078	Low *κ*
							0.153	wt
							0.431	High *κ*
NFM	136	1	3.40	0.390	−0.051	2.54	0.037	Low *κ*
							0.134	wt
							0.516	High *κ*
PNT4	114	13	3.26	0.298	0.018	2.54	0.044	Low *κ*
							0.213	wt
							0.421	High *κ*

**Table 2:** Hydrodynamic radii (*R_h_*) and average solvent accessible surface area (*SASA*) of the three model proteins and their derived *κ* variants. Mean values and standard deviations from three independent measurements are reported. Volume, surface area and flattening indices of the ellipsoids were derived from the model proposed by Wu and co-authors [23].

Protein Variant		*R_h_* (nm)	*SASA* (nm^2^)	Volume (nm^3^)	*f_b_* *	*f_c_* *
N_TAIL_	Low *κ*	2.78 ± 0.03	113.2 ± 2.1	49.5 ± 2.2	0.26 ± 0.02	0.41 ± 0.16
	wt	2.73 ± 0.03	105.6 ± 1.4	46.3 ± 1.8	0.25 ± 0.01	0.39 ± 0.16
	High *κ*	2.58 ± 0.05	89.3 ± 1.0	37.5 ± 2.3	0.24 ± 0.02	0.38 ± 0.16
NFM	Low *κ*	3.37 ± 0.05	136.2 ± 2.0	99.1 ± 5.0	0.14 ± 0.02	0.23 ± 0.12
	wt	3.31 ± 0.04	124.5 ± 2.4	93.2 ± 4.8	0.12 ± 0.02	0.19 ± 0.10
	High *κ*	3.05 ± 0.10	81.5 ± 0.4	69.7 ± 8.0	-0.03 ± 0.08	0.02 ± 0.09
PNT4	Low *κ*	2.39 ± 0.04	106.8 ± 3.0	28.1 ± 2.1	0.42 ± 0.03	0.53 ± 0.13
	wt	2.36 ± 0.05	106.8 ± 2.5	26.8 ± 2.1	0.44 ± 0.03	0.54 ± 0.12
	High *κ*	2.43 ± 0.05	69.0 ± 1.0	29.9 ± 2.4	0.19 ± 0.03	0.31 ± 0.15

* flattening indices relative to *b* (1-*b*/*a*) and *c* (1-*c*/*a*) axis.

## Data Availability

Not applicable.

## Supplementary Materials

The following supporting information can be downloaded at: https://www.mdpi.com/article/10.3390/biom12040561/s1: Table S1: Size of semi-axes of ellipsoids; Figure S1: Amino acid sequences of model IDPs; Figure S2: Far-UV CD spectra of model IDPs; Figure S3: Native MS analyses of model IDPs; Figure S4: Compactness of conformational components of model IDPs studied by native MS; Figure S5: Relative abundance of conformational components of model IDPs studied by native MS; Figure S6: NanoESI-MS spectra of model IDPs under conditions affecting electrostatic interactions.
