# Supplementary material for: Distribution of Charged Residues Affects the Average Size and Shape of Intrinsically Disordered Proteins
Source: Biomolecules. 2022 Apr 9;12(4):561. doi: 10.3390/biom12040561 (PMC9031945; doi:10.3390/biom12040561)
Supplement: Supplementary file 1 [file biomolecules-12-00561-s001.zip › biomolecules-1646632-supplementary.pdf]

SUPPLEMENTARY MATERIAL FOR:

Distribution of charged residues affects the average size and shape of intrinsically disordered proteins

Greta Bianchi[1], Marco Mangiagalli[1], Alberto Barbiroli[2], Sonia Longhi[3], Rita Grandori[1], Carlo Santambrogio[1*], Stefania Brocca[1*]

[1]Dept. of Biotechnology and Biosciences, University of Milano-Bicocca, 20126, Milan, Italy

[2]Dept. of Food, Environmental and Nutritional Sciences, University of Milan, 20133, Milan, Italy

[3]Lab. Architecture et Fonction des Macromolécules Biologiques (AFMB), UMR 7257, Aix-Marseille University and CNRS, 13288, Marseille, France

[*] Correspondence:

Prof. Stefania Brocca, Department of Biotechnology and Biosciences, University of Milano-Bicocca, Piazza della Scienza 2, 20126 Milan, Italy.

Phone: 0264483518

E-mail: stefania.brocca@unimib.it;

Dr. Carlo Santambrogio, Department of Biotechnology and Biosciences, University of Milano-Bicocca, Piazza della Scienza 2, 20126 Milan, Italy.

Phone: 0264483363

E-mail: carlo.santambrogio@unimib.it

Table S1. Size of the semi-axes of ellipsoids assimilated to the model proteins studied in this work

Protein variant		*a* (nm)	*b* (nm)	*c* (nm)	Surface* (nm^2)
N_{TAIL}	Low κ	3.00 ± 0.03	2.21 ± 0.03	1.78 ± 0.04	67.5 ± 1.9
	wt	2.90 ± 0.02	2.17 ± 0.03	1.76 ± 0.03	64.4 ± 1.5
	High κ	2.67 ± 0.01	2.02 ± 0.04	1.66 ± 0.06	55.8 ± 2.1
NFM	Low κ	3.29 ± 0.02	2.83 ± 0.04	2.54 ± 0.07	104.5 ± 3.4
	wt	3.15 ± 0.03	2.78 ± 0.04	2.54 ± 0.07	100.0 ± 3.3
	High κ	2.55 ± 0.01	2.62 ± 0.02	2.49 ± 0.09	81.9 ± 6.2
PNT4	Low κ	2.92 ± 0.04	1.69 ± 0.07	1.36 ± 0.03	48.1 ± 2.3
	wt	2.91 ± 0.04	1.64 ± 0.08	1.34 ± 0.03	46.8 ± 2.4
	High κ	2.34 ± 0.02	1.89 ± 0.05	1.61 ± 0.07	47.4 ± 2.3

* calculated by Thomsen's approximation (maximal discrepancy ~1%)

N_{TAIL}

```
Low-k     MHHHHHHTTERDISRAVGPDQAQVSFLHGKQSENRLPELGGKEKERVDQSRGEARESYED   60
wt        MHHHHHHTTEDKISRAVGPRQAQVSFLHGDQSENELPRLGGKEDRRVKQSRGEARESYRE   60
High-k    MHHHHHHTTRKRISRAVGPRQAQVSFLHGRQSRNRLPRLGGKRKRRVRQSRGRADESYED   60

Low-k     TGPSRASDARAAHLPTGTPLDIDTASRSSQDPQRSERSADALLRLQAMAGISERQGSDTD   120
wt        TGPSRASDARAAHLPTGTPLDIDTASESSQDPQDSRRSADALLRLQAMAGISEEQGSDTD   120
High-k    TGPSEASDAEAAHLPTGTPLDIDTASESSQDPQDSEESADALLELQAMAGISEDQGSDTD   120

Low-k     TPIVYNDRNLLDGS   134
wt        TPIVYNDRNLLDGS   134
High-k    TPIVYNDENLLDGS   134
```

NFM

```
Low-k     MHHHHHHDGAKGSDKEKIAVNGEVEGKEEVKQETKEKGSGDEREKGVVTNGLDLSPAKEK   60
wt        MHHHHHHKGAKGSRKEDIAVNGEVEGKEEVEQETKEKGSGREEEKGVVTNGLDLSPADEK   60
High-k    MHHHHHHKGAKGSRKKKIAVNGKVKGKKKVKQKTKKKGSGRKKKKGVVTNGLKLSPAKED   60

Low-k     EGGDKSEEKVVVTETVEKITSEGGRGATEYITKSVTVTQEVKEHKETFKEKLVSTEKVEK   120
wt        KGGDKSEEKVVVTKTVEKITSEGGDGATKYITKSVTVTQKVEEHEETFEEKLVSTKKVEK   120
High-k    DGGDESEEEVVVTETVEEITSEGGDGATEYITESVTVTQEVEEHEETFEEELVSTEEVED   120

Low-k     VTSHAIVEKVTQSDGS   136
wt        VTSHAIVKEVTQSDGS   136
High-k    VTSHAIVEEVTQSDGS   136
```

PNT4

```
Low-k     MHHHHHHKETPRVDRDKSLMQDSCKEGGVPERLPMLSREFRCSGSDRPIIQELREKGSHP   60
wt        MHHHHHHEETPDVRRKDSLMQDSCKRGGVPKRLPMLSEEFECSGSDDPIIQELEREGSHP   60
High-k    MHHHHHHKRTPRVRRKKSLMQRSCKRGGVPKRLPMLSRKFRCSGSKRPIIQRLEDEGSHP   60

Low-k     GGSLDLREPPQSSGNSRNQPDRQLKTGDAASPGGVQRPGTPMPKSEIMPIKEGS   114
wt        GGSLRLREPPQSSGNSRNQPDRQLKTGDAASPGGVQRPGTPMPKSRIMPIKKGS   114
High-k    GGSLELEEPPQSSGNSENQPDEQLDTGDAASPGGVQEPGTPMPDSEIMPIDDGS   114
```

Figure S1. Amino acid sequences of model IDPs. The wt, low-κ and high-κ variants within each of the three sets have been aligned to highlight the sequence design. Positively charged residues are shown in blue, negatively charged residues in red, yellow highlights proline residues. Sequence alignments were carried out with Clustal Omega (Sievers et al, 2011, doi:10.1038/msb.2011.75)

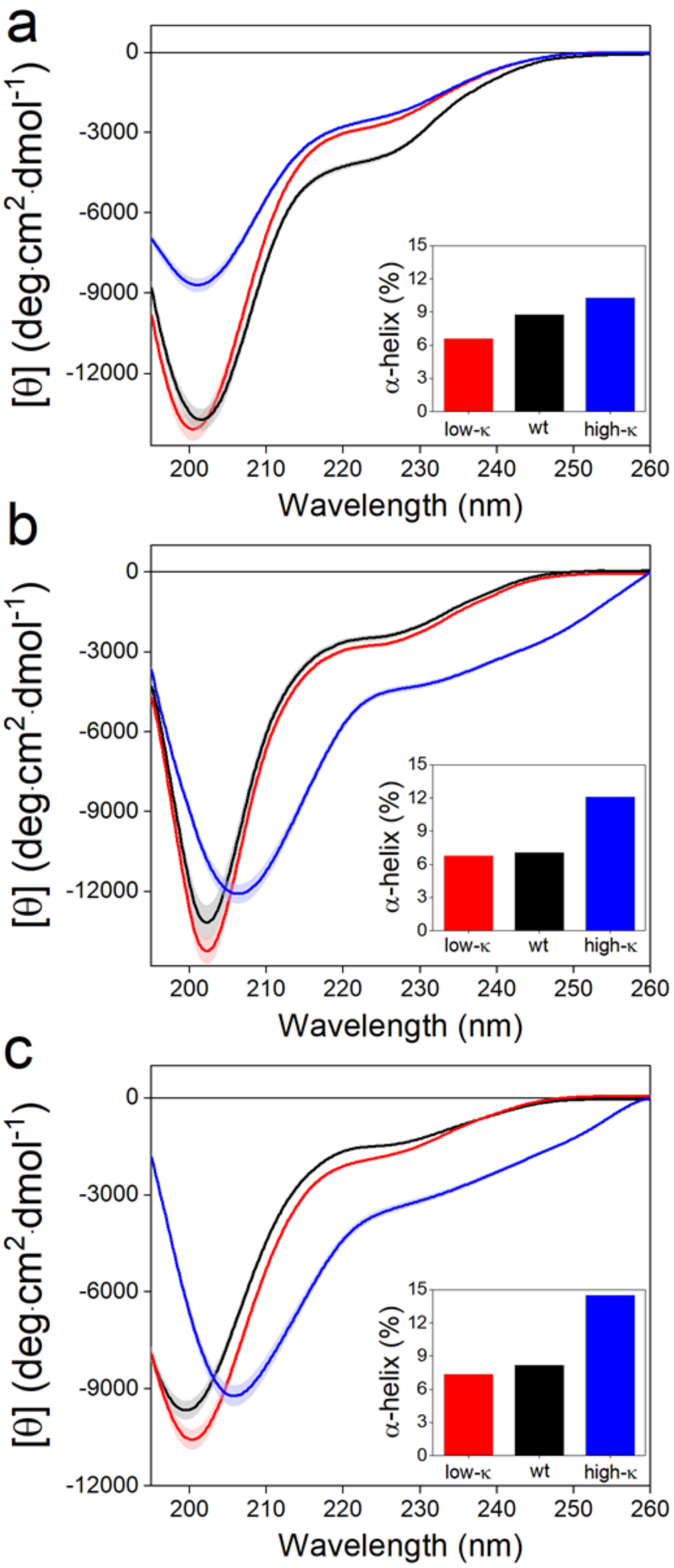

Figure S2. Far-UV CD spectra of model IDPs. CD spectra of N_{TAIL} (**a**), NFM (**b**), and PNT4 (**c**) variants. CD analyses were performed in PBS. The shadowed area refers to the standard deviation of three independent measurements. In black the spectrum of wt proteins, while red and blue are used for the spectra of low-*κ* and high-*κ* permutants, respectively. Inset of each panel: percentage of α-helical content as estimated by BeStSel software (Micsonai et al., 2018; doi:10.1093/nar/gky497).

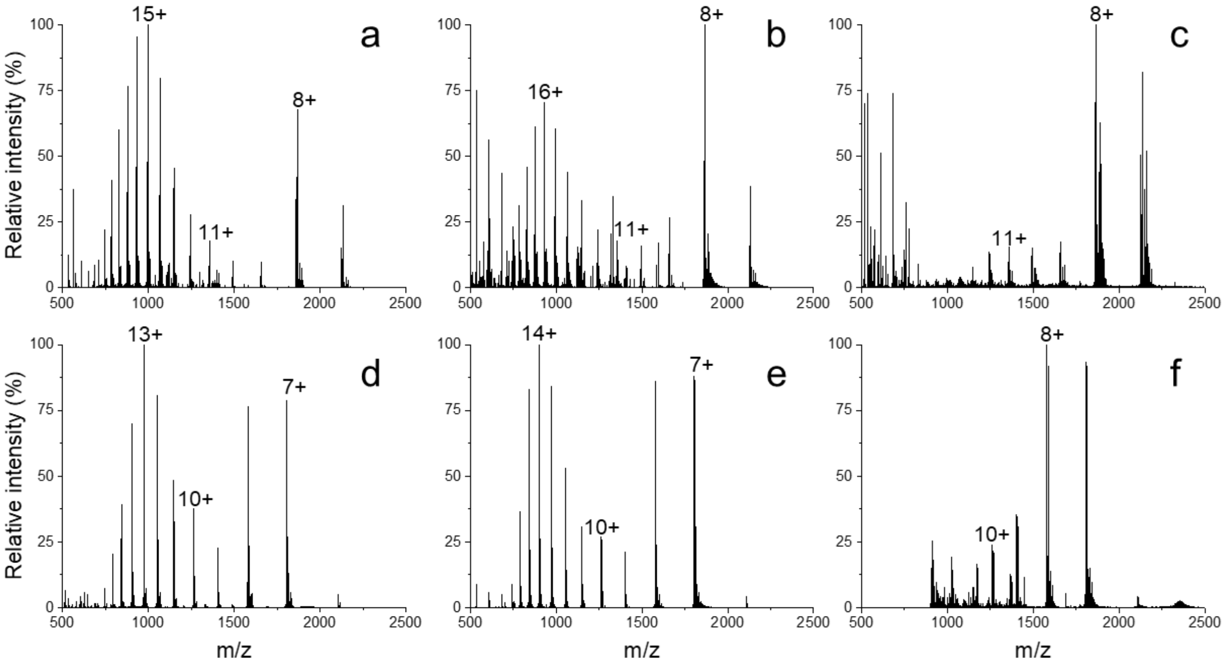

Figure S3. Native MS analyses of model IDPs. NanoESI-MS spectra of NFM (**a**-**c**) and PNT4 (**d**-**f**) variants acquired under non-denaturing conditions (50 mM ammonium acetate, pH 7.0). Low-*κ* variants (**a**, **d**), wt variants (**b**, **e**) and high-*κ* variants (**c**, **f**) are reported.

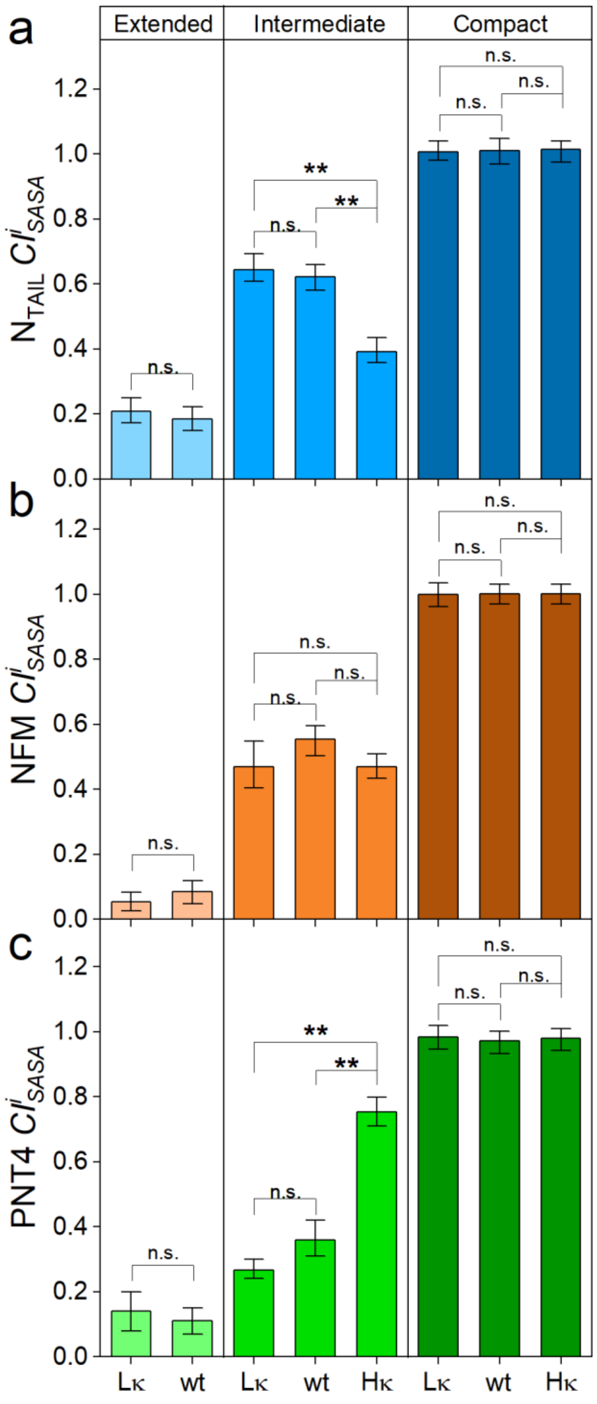

Figure S4. Compactness of conformational components of model IDPs studied by native MS. Bar chart reporting CI^{i}_{SASA} of NTAIL (**a**), NFM (**b**) and PNT4 (**c**). Mean values of three independent measurements are shown with error bars indicating standard deviations. Statistical analyses were carried out using Welch's t-test (n.s.: p >0.05, *: p < 0.05, **: p< 0.01).

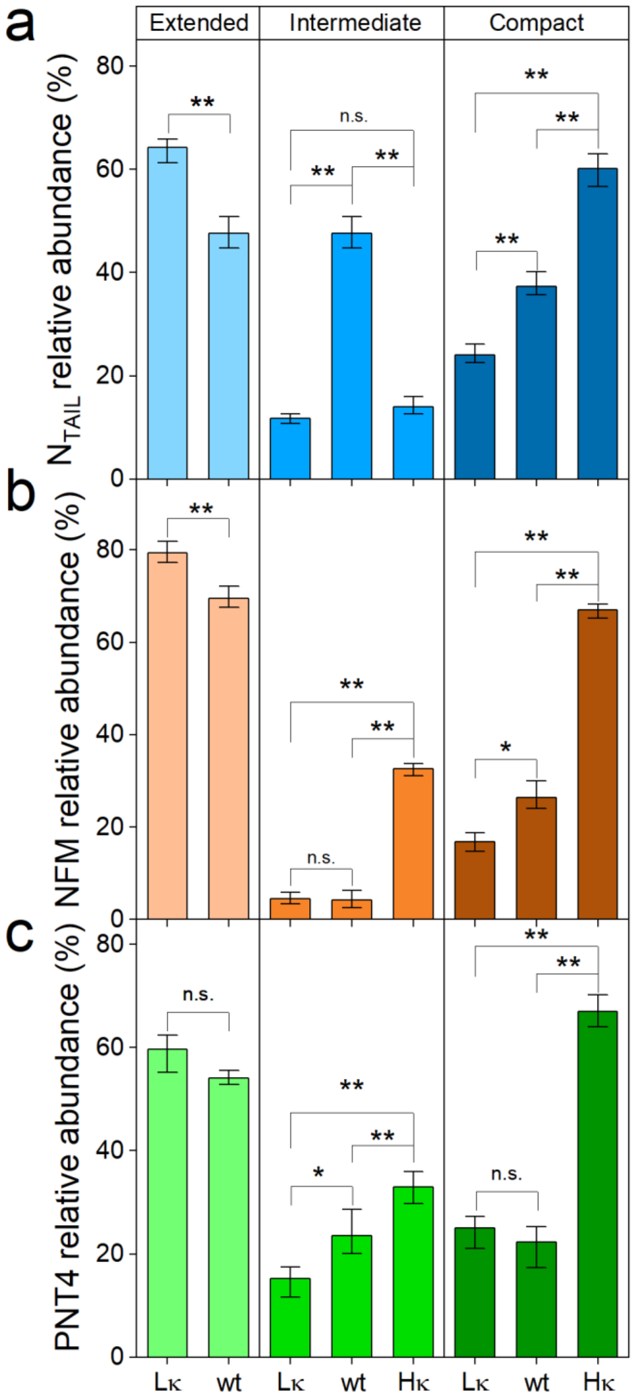

Figure S5. Relative abundance of conformational components of model IDPs studied by native MS. Bar chart reporting the relative abundance of single conformational components (extended; intermediate; compact) of N_{TAIL} (**a**), NFM (**b**) and PNT4 (**c**). Mean values of three independent measurements are represented with error bars indicating standard deviations. Statistical analyses were carried out using Welch's t-test (p >0.05 n.s., * p < 0.05, **p< 0.01).

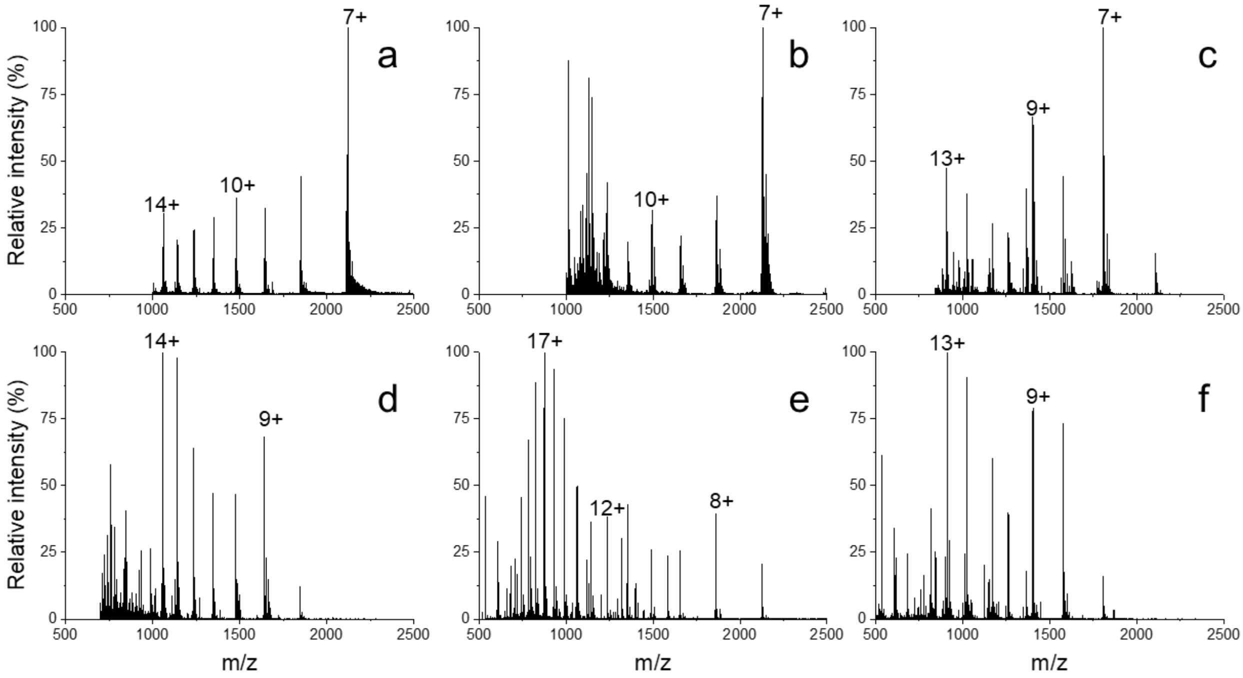

Figure S6. NanoESI-MS spectra of model IDPs under conditions affecting electrostatic interactions. NanoESI-MS spectra of high-κ variants of N_{TAIL} (**a**, **d**), NFM (B, E) and PNT4 (C, F) acquired in the presence of 200 mM ammonium acetate pH 7.0 (**a-c**), and in the presence of 1% formic acid (**d-f**).
